# Improving the Therapeutic Efficacy of Sorafenib for Hepatocellular Carcinoma by Repurposing Disulfiram

**DOI:** 10.3389/fonc.2022.913736

**Published:** 2022-07-14

**Authors:** Gong Zhang, Yufeng Wang, Bryan C. Fuchs, Wei Guo, David L. Drum, Derek J. Erstad, Baomin Shi, Albert B. DeLeo, Hui Zheng, Lei Cai, Liyuan Zhang, Kenneth K. Tanabe, Xinhui Wang

**Affiliations:** ^1^ Division of Gastrointestinal and Oncologic Surgery, Department of Surgery, Massachusetts General Hospital, Harvard Medical School, Boston, MA, United States; ^2^ Department of Hepatobiliary and Pancreatic Surgery, The First Affiliated Hospital of Zhengzhou University, Zhengzhou, Henan, China; ^3^ Department of General Surgery, Tongji Hospital, School of Medicine, Tongji University, Shanghai, China; ^4^ Biostatistics Center, Massachusetts General Hospital, Harvard Medical School, Boston, MA, United States

**Keywords:** sorafenib, disulfiram, copper, hepatic cancer stem cells, ERK pathway

## Abstract

**Background:**

Sorafenib, a kinase inhibitor, is a standard treatment for advanced hepatocellular carcinoma (HCC) but provides only a limited survival benefit. Disulfiram (DSF), a drug for treating alcoholism and a chelator of copper (Cu), forms a complex with Cu (DSF/Cu). DSF/Cu is a potent inducer of autophagic apoptosis of cancer stem cells, which can demonstrate drug resistance. Thus, we hypothesized that DSF/Cu could increase the sensitivity of HCC cells to sorafenib by targeting hepatic cancer stem cells.

**Methods:**

The synergistic effect of DSF/Cu and sorafenib on human HCC cell lines was assessed by cell viability MTT assay. Changes in stemness gene expression in HCC cells were investigated by assessing the presence of hepatic cancer stem cells (HCSCs) (defined as ALDH^+^ cells) using flow cytometry, sphere formation ability as an index of *in vitro* tumorigenicity, and expression of stemness gene-encoded proteins by western blot. Autophagic apoptosis and the ERK signaling pathway were also assessed by western blot. Most importantly, the *in vivo* anti-tumor efficacy of DSF/Cu and sorafenib was tested using orthotopic HCC xenografts in mice.

**Results:**

Compared with sorafenib alone, DSF/Cu + sorafenib synergistically inhibited proliferation of all HCC cell lines, decreased the stemness of HCC cells, and increased the autophagy and apoptosis of HCC cells. The mechanism by which DSF/Cu mediated these phenomena with sorafenib was sustained activation of the ERK pathway. The combination of DSF/Cu (formed with endogenous Cu^2+^) and sorafenib was significantly more effective than sorafenib alone in inhibiting the growth of orthotopic HCC xenografts in mice. This *in vivo* anti-tumor efficacy was associated with decreased stemness in treated HCC tumors.

**Conclusions:**

DSF/Cu and sorafenib can synergistically and effectively treat HCC by targeting HCSCs *in vitro* and *in vivo*. Our data provide a foundation for clinical translation.

## Introduction

Primary liver cancer is the seventh most common malignancy and the fourth leading cause of cancer death worldwide with high rates of incidence (4.7%) and mortality (8.2%) (1, 2). Up to 75-85% of primary liver cancers are hepatocellular carcinoma (HCC) (1). Surgical interventions, including partial hepatectomy, liver transplantation, transarterial chemoembolization, and ablation, are effective in patients with early-stage HCC (3). However, most patients with HCC are diagnosed in the advanced stage, past the optimal time for surgical treatment (4). Therefore, systemic pharmacotherapy and radiotherapy play an important role in patients with advanced HCC, although they do not markedly improve the overall survival rate.

No effective drugs for treating advanced HCC were available until 2007, when sorafenib, a multi-kinase inhibitor, was approved for use, opening a new era in the treatment of advanced HCC. Sorafenib exerts its anti-tumor ability by blocking the RAF/MEK/ERK pathway, vascular endothelial growth factor receptor (VEGFR), and platelet-derived growth factor receptor (PDGFR) (5). Sorafenib prolongs median survival time by 2-3 months and improves the quality of life of patients with advanced HCC (6, 7). However, few HCC patients respond to sorafenib and rapidly become treatment-resistant, resulting in disease progression (8).

In recent years, considerable evidence indicates that hepatic cancer stem cells (HCSCs), the subpopulation of HCC cells considered responsible for HCC initiation, metastasis, and recurrence, are highly resistant to therapy (9, 10). HCSCs possess the characteristics of self-renewal (11), stemness gene expression, and elevated expression of aldehyde dehydrogenase (ALDH^+^), distinguishing them from bulk tumor cells that are essentially ALDH^-^. HCSCs (12, 13) also express an array of cell surface markers including CD133, CD90, CD24, EpCAM, CD44, OV6, and CD13 (14). ALDH^+^ HCC cells contribute to chemoresistance and are associated with a higher rate of metastasis than ALDH^-^ HCC cells (13). As acquired resistance to sorafenib in HCC patients is closely correlated with HCSCs (10, 15, 16), the use of therapies that target HCSCs in combination with sorafenib could improve the efficacy of sorafenib treatment for advanced HCC.

Disulfiram (DSF), a drug used for treating alcoholism since 1951, is an ALDH inhibitor. DSF binds with copper ions (Cu^2+^) to form DSF/Cu complexes that have anti-tumor efficacy (17). We previously found that pancreatic cancer stem cells (CSCs) and non-stem cells (non-CSCs) are effectively targeted by incorporating DSF/Cu into standard chemoradiation regimens (18). Here, we investigated whether treatment with DSF/Cu increases the sensitivity of HCC cells, in particular HCSCs, to sorafenib in both *in vitro* and *in vivo* preclinical experiments.

HCSCs present in four well-established human HCC cell lines, HepG2, Hep3B, SNU423, and SNU387, were identified by flow cytometry as ALDH^+^ HCC cells (12, 13). Independently of ALDH expression, HCSCs were also identified through the sphere formation of single tumor cells, a surrogate marker for the CSC-associated activity of self-renewal, as well as expression of the stemness genes SOX9 (19), HER2 (20, 21), and c-Myc (22). The MEK/ERK signaling pathway was examined in cell lines treated with a combination of DSF/Cu + sorafenib to monitor its impact on therapeutic efficacy (23). Lastly, the effect of DSF/Cu and/or sorafenib on the growth of HCC cells was examined *in vivo* in an orthotopic HepG2-derived xenograft mouse model.

## Materials and Methods

### Cell Culture

The human HCC cell lines HepG2, Hep3B, SNU387, and SNU423 were obtained from ATCC. HepG2 and Hep3B cell lines were cultured in Dulbecco’s Modified Eagle’s Medium (DMEM; Mediatech, Inc.) supplemented with 10% fetal bovine serum (FBS; Atlanta Biologicals). SNU387 and SNU423 cell lines were cultured in RPMI 1640 medium (Corning) with 10% FBS. All cell lines were cultured at 37°C in a humidified atmosphere of 5% CO_2_.

### Chemical Reagents, Antibodies, and Monoclonal Antibodies

Tetraethylthiuram disulfide (disulfiram, DSF), copper (II) D-gluconate(C_12_H_22_CuO_14_) or Copper (II) chloride (CuCl_2_), and MEK inhibitor U0126 were purchased from Sigma-Aldrich (St. Louis, MO, USA). Sorafenib was obtained from Bayer Corporation (Whippany, NJ, USA). DSF, sorafenib, and U0126 were reconstituted in DMSO for all *in vitro* experiments. DSF was reconstituted in olive oil for *in vivo* experiments. Copper was reconstituted in distilled water for all experiments. ALDH^+^ cells were determined by ALDH activity measured by ALDEFLUOR^®^ reagent (Stem Cell Technologies, Cambridge, MA, USA).

Antibodies (Ab) and monoclonal Ab (mAb) and their dilutions used for western blotting were specific rabbit mAbs for human ERK1/2 (#4695, 1:1000), human MEK1/2 (#9126, 1:1000), human phosphorylated ERK1/2 (#9101, 1:1000), human phosphorylated MEK1/2 (#9154, 1:1000), human HER2/ERBB2 (#2165, 1:1000), human c-Myc (#9402, 1:1000), LC3A/B (#4108, 1:1000), cleaved PARP (#5625, 1:1000), human β-actin (#4970, 1:2000), specific rabbit mAbs, and goat anti-rabbit IgG HRP-conjugated antibody (#7074, 1:2000), purchased from Cell Signaling Technology (Danvers, MA, USA). Human SOX9-specific rabbit Ab (ab26414, 1 μg/mL) was purchased from Abcam (Cambridge, MA, USA). All ab were diluted in Tris-buffered saline with 0.1% Tween^®^ 20 (TBST) containing 5% non-fat dry milk plus 2% bovine serum albumin (BSA). All dilutions were prepared immediately before use.

### Animals

Male NSG mice at 6 weeks of age were obtained from the Massachusetts General Hospital COX7 animal facility. The Institutional Animal Care and Use Committee approved all animal experiments.

### MTT Assay and Synergy Analysis

HCC cells were plated in 96-well plates at a density of 5000 cells/well in 100 μL appropriate complete medium and incubated overnight. Cells were then treated with the indicated concentrations of drugs for 48 h before determining cell viability using 3-(4,5-dimethylthiazol-2-yl)-2,5-diphenyltetrazolium bromide (MTT) assay (Sigma). The synergistic effect of drug combinations was evaluated using the Chou-Talalay method with CompuSyn software (www.combosyn.com) to calculate combination index (CI) values. CI values of <1, = 1, and >1 indicated synergistic, additive, and antagonistic effects, respectively (24).

### Flow Cytometry

Cells were seeded in 6-well plates at a density of 2×10^5^ cells/well in 2 mL appropriate complete medium and incubated overnight. Cells were then treated with DMSO, DSF/Cu, sorafenib, or DSF/Cu + sorafenib for 12 h, and IC_50_ values (**Table 1**) of DSF/Cu and sorafenib for each cell line were used for experiments. For the detection of ALDH^+^ activity in each cell line, flow cytometry was performed as previously described (18).

**Table 1:** IC_50_ of sorafenib and DSF/Cu in HCC cell lines *in vitro*. Data are shown as mean ± SD (n=3).

HCC cell lines	IC_50_ (μM/1 μM)	IC_50_ (μM)
	DSF/Cu	Sorafenib
HepG2	0.236 ± 0.024	24.550 ± 3.286
Hep3B	0.117 ± 0.015	9.599 ± 2.252
SNU387	0.033 ± 0.008	25.210 ± 0.939
SNU423	0.247 ± 0.032	21.990 ± 1.881

### Sphere Formation Assay

Cells were plated in 24-well plates at a density of 1000 cells/well in 0.5 mL appropriate complete medium followed immediately by treatment with DMSO, DSF, Cu, DSF/Cu, sorafenib, or DSF/Cu + sorafenib for an additional 24 h. IC_50_ values (**Table 1**) of DSF and sorafenib in each cell line were used for experiments. Sphere formation procedures were performed as previously described (18). For secondary/tertiary sphere formation experiments, primary/secondary spheres were dissociated by tumor/tissue dissociation reagent (BD Biosciences, San Jose, CA, USA) into single cells followed by the same sphere formation procedure. Sphere formation was performed using cells from a single cell suspension collected from HCC cell lines or disaggregated (with collagenase IV, 1 mg/mL in PBS) mouse tumors.

### Western Blot Analysis

Cells were plated in 6-well plates at a density of 1×10^5^ cells/well in 2 mL appropriate complete medium and incubated overnight. All cells were treated and collected at time point(s) as indicated. Cells were collected and lysed in lysis buffer (10 mM Tris-HCl (pH 8.2), 1% NP40, 1 mM EDTA, 0.1% BSA, 150 mM NaCl) containing 1/50 (vol/vol) protease inhibitor cocktail (Calbiochem, Burlington, MA, USA). Western blotting to assess protein levels of stemness genes was carried out as previously described (25).

### Orthotopic HCC Xenograft Mouse Model

Orthotopic mouse models were established by inoculating 6×10^5^ HepG2 cells/mouse in the left hepatic lobe of 6-week-old male NSG mice. Surgical procedures were performed under general anesthesia using maintenance inhalational isoflurane anesthesia (2% (v/v) in 1 L/min O_2_). An 8-10 mm transverse incision was made below the xiphoid and perpendicular to the median line after sterilization with 70% ethanol and povidone-iodine solution. A cotton-tipped applicator was used to expose and stabilize the left hepatic lobe. A total volume of 30 μL cell suspension was injected into the left hepatic lobe using a 27G 1/2 insulin syringe needle. After the needle was removed from the liver, a cotton-tipped applicator was placed over the puncture site with gentle pressure for 30 seconds to prevent leakage of the tumor cell suspension and achieve complete hemostasis. The skin was cleaned with 70% alcohol, and the wound was sutured with a Plus 5-0 suture line. Orthotopic HCC xenograft tumor formation was established on day 4 based on our preliminary experiments.

### *In Vivo* Anti-Tumor Efficacy

Mice were divided into four groups (n=5 per group) using a randomization strategy. Mice were either (1) untreated, (2), treated with oral administration of sorafenib (30 mg/kg/day) for 12 days, (3) treated with oral administration of sorafenib (30 mg/kg/day) for 12 days and intraperitoneal (i.p.) administration of DSF (25 mg/kg in olive oil) twice per week, or (4) treated with sorafenib and DSF along with i.p. administration of copper (II) D-gluconate (1 mg/kg) twice per week. All oral administrations were given by oral gavage using an 18-gauge plastic feeding tube (Solomon Scientific, San Antonio, TX, USA). Body weight was measured every 3 days. On day 24, all mice were euthanized, and tumor volume was measured, and tumors were collected for sphere formation assay.

### Statistical Analysis

For both *in vivo* and *in vitro* data, the differences among three or more groups were determined using one-way ANOVA followed by Tukey’s method to adjust for multiple comparisons. Additional two-way ANOVA was adopted as specified. All data are expressed as mean ± standard deviation (SD) unless specified otherwise. Data were analyzed and graphs were plotted using GraphPad Prism 8 software. Results were obtained from two or three independent experiments. Differences between groups were considered significant when p<0.05.

## Results

### DSF/Cu and Sorafenib Synergistically Inhibit the Growth of HCC Cells *In Vitro*

**Figure 1:**
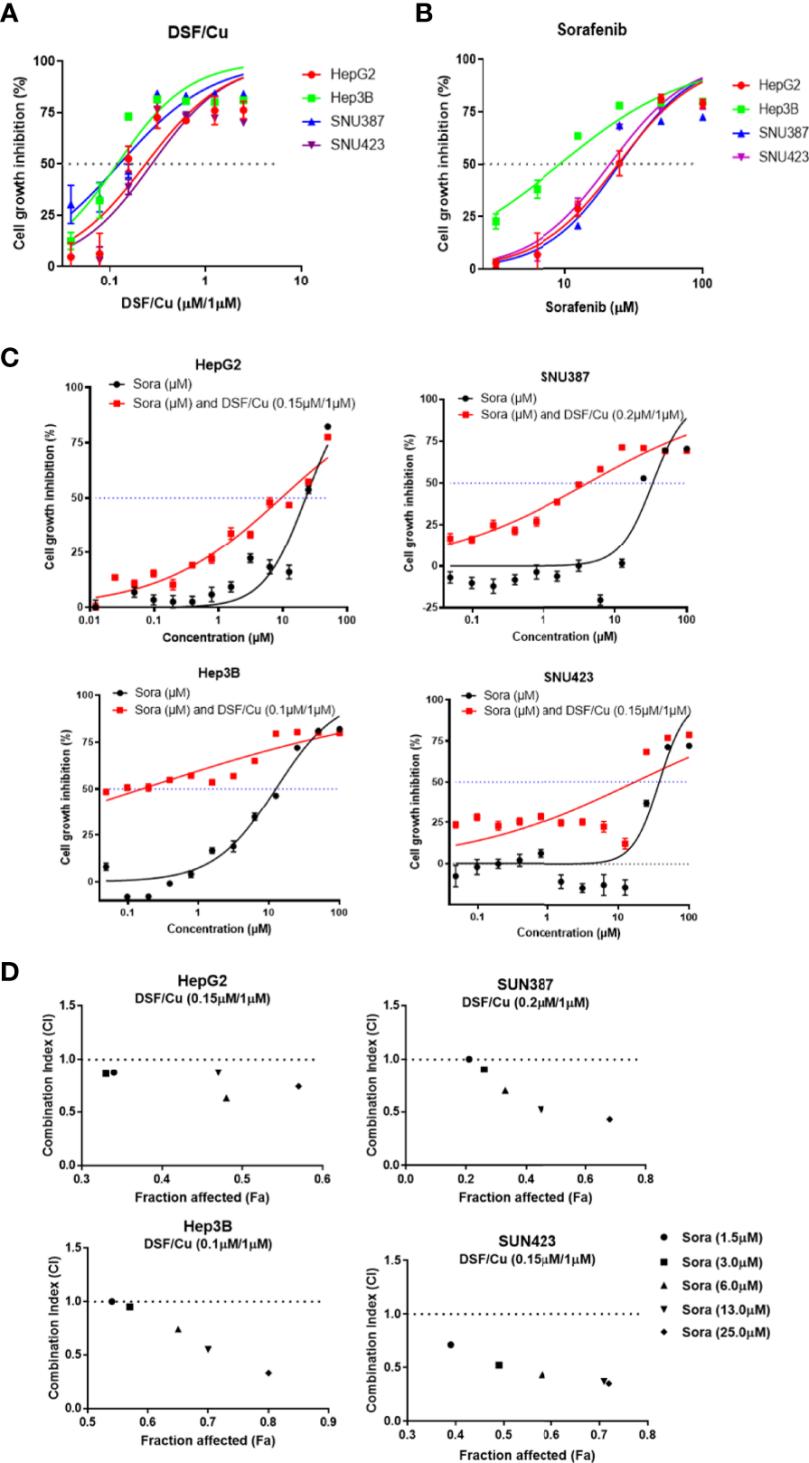
DSF/Cu and sorafenib (or Sora) synergistically inhibit the growth of HCC cells *in vitro*. The HCC cell lines HepG2, Hep3B, SNU387, and SNU423 were treated with DSF/Cu or sorafenib at different concentrations for 48 h followed by MTT assay to evaluate their cell growth inhibition rate **(A, B)**. Cells were treated with a fixed concentration of DSF/Cu [HepG2 (0.15 μM/1 μM), Hep3B (0.10 μM/1 μM), SNU387 (0.20 μM/1 μM), and SNU423 (0.15 μM/1 μM)] and different concentrations of sorafenib for 48 h. Cell growth inhibition was determined by MTT assay **(C)**. CI values were calculated using CompuSyn software and graphed for each cell line **(D)**. CI<1, = 1, and >1 indicated synergistic, additive, and antagonistic effects, respectively. IC_50_, half-maximal inhibitory concentration. All experiments were performed in triplicate for each cell line.

Four HCC cell lines—HepG2, Hep3B, SNU387, and SNU423—were used to evaluate the sensitivity of HCC cells to DSF/Cu and/or sorafenib. Cells were treated with a gradient of DSF/Cu concentrations or sorafenib alone for 48 h followed by determination of cell growth inhibition using MTT assay. A fixed dose of 1 μM Cu^2+^ was used with DSF in these experiments based on our previous experience and related publications (26, 27). DSF/Cu or sorafenib alone suppressed the growth of all four HCC cell lines in a dose-dependent manner (**Figures 1A, B**). The IC_50_ values of DSF/Cu and sorafenib alone for the four HCC cell lines are presented in **Table 1**.

To determine whether DSF/Cu and sorafenib could act synergistically to inhibit the growth of HCC cells, each HCC cell line was treated with DSF/Cu at a fixed concentration that was lower than the IC_50_ DSF/Cu value for that cell line and various concentrations of sorafenib for 48 h. Combining DSF/Cu with sorafenib significantly increased the inhibition of cell growth compared with sorafenib alone (**Figure 1C**). Fraction affected values, namely, the fraction of cells inhibited by drugs, were obtained for each HCC cell line. The CI value was calculated from the fraction affected value of each drug combination. At combinations below IC_50_ values, DSF/Cu showed synergistic interactions (CI<1) with sorafenib at indicated doses in inhibiting the growth of all HCC cell lines (**Figure 1D**).

### HCSCs Are Sensitive to DSF/Cu and Sorafenib

**Figure 2:**
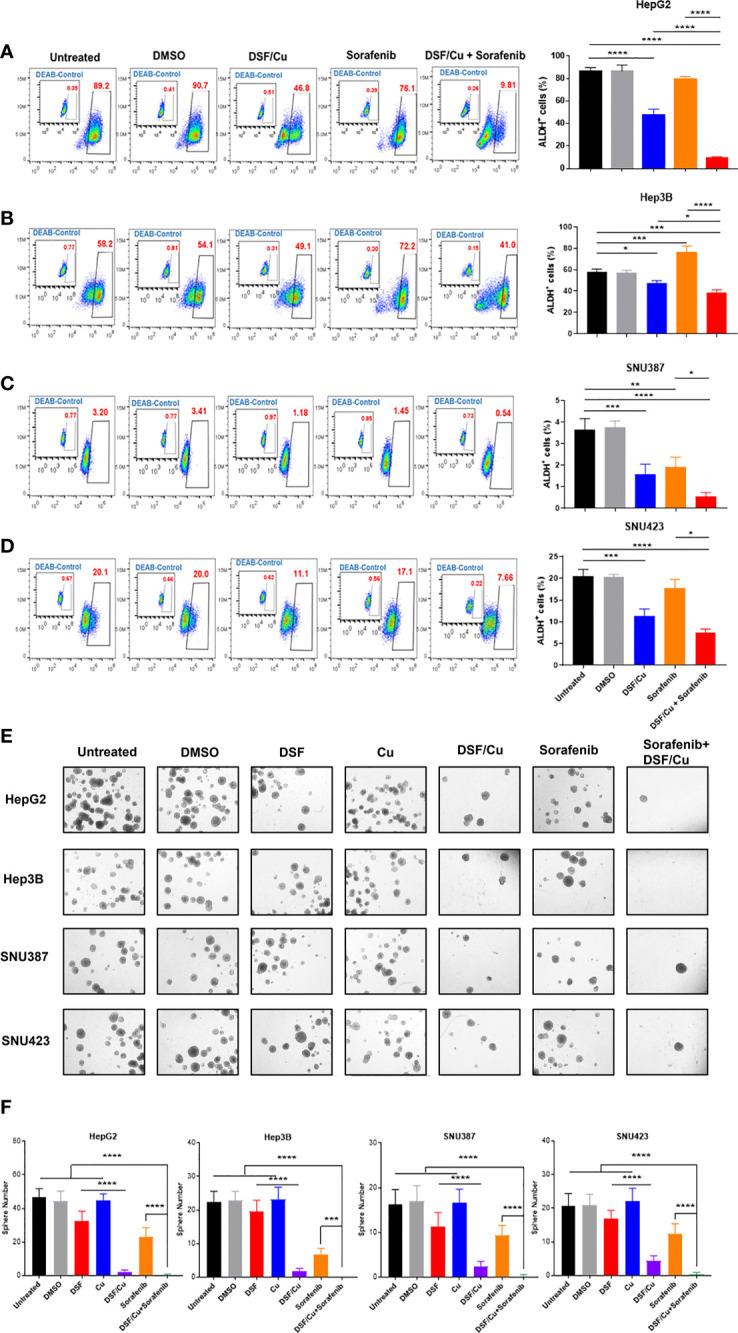
HCSCs are sensitive to DSF/Cu and sorafenib. The HCC cell lines HepG2 **(A)**, Hep3B **(B)**, SNU387 **(C)**, and SNU423 **(D)** were treated as indicated, and ALDH^+^ cells was measured using flow cytometry. Sphere formation assays were performed in six-well plates by seeding treated cells and culturing for 14 days. Spheres were quantified by counting sphere numbers per well on day 14 **(E, F)**. All experiments were performed in triplicate for each cell line, and data are shown as mean ± SD. *p<0.05, **p<0.01, ***p<0.001, ****p<0.0001.

ALDH^+^ HCSCs in all four HCC cell lines were identified by flow cytometry (**Figures 2A–D**). DSF/Cu treatment reduced the percentage of ALDH^+^ cells in all cell lines (**Figures 2A–D**). In the Hep3B cell line, the percentage of ALDH^+^ cells increased after sorafenib treatment (p=0.0003) (**Figure 2B**). However, sorafenib had little impact on the percentage of ALDH^+^ cells in HepG2 and SNU423 cell lines (p=0.2558 and 0.2324, respectively) (**Figures 2A–D**). Notably, DSF/Cu + sorafenib was significantly more effective in reducing ALDH^+^ cells in all four HCC cell lines compared with either drug alone (**Figures 2A–D**).

A critical characteristic of CSCs is their ability to self-renew, which was assessed using sphere formation assay. As monotherapy, DSF/Cu was more effective than sorafenib in preventing the formation of spheres in HepG2 (p<0.001), Hep3B (p=0.0345), SNU387 (p=0.0011), and SNU423 (p<0.001) cells (**Figures 2E, F**). Notably, DSF/Cu + sorafenib resulted in significantly less sphere formation than any single treatment alone (p<0.001) except for DSF/Cu alone (p>0.05) (**Figures 2E, F**). By comparing sphere numbers of DSF/Cu vs. DSF treated cells in all 4 cell lines, it is clear that the effect of DSF on targeting HCSCs is Cu-dependent (**Figures 2E, F**). To determine whether DSF/Cu and sorafenib effects were interrelated, we statistically assessed the interaction effect between DSF/Cu and sorafenib using two-way ANOVA and found that their interaction effect was significant (p<0.0001 in all four cell lines).

### DSF/Cu and Sorafenib Decrease Stemness Gene Expression in HCC Cells

**Figure 3:**
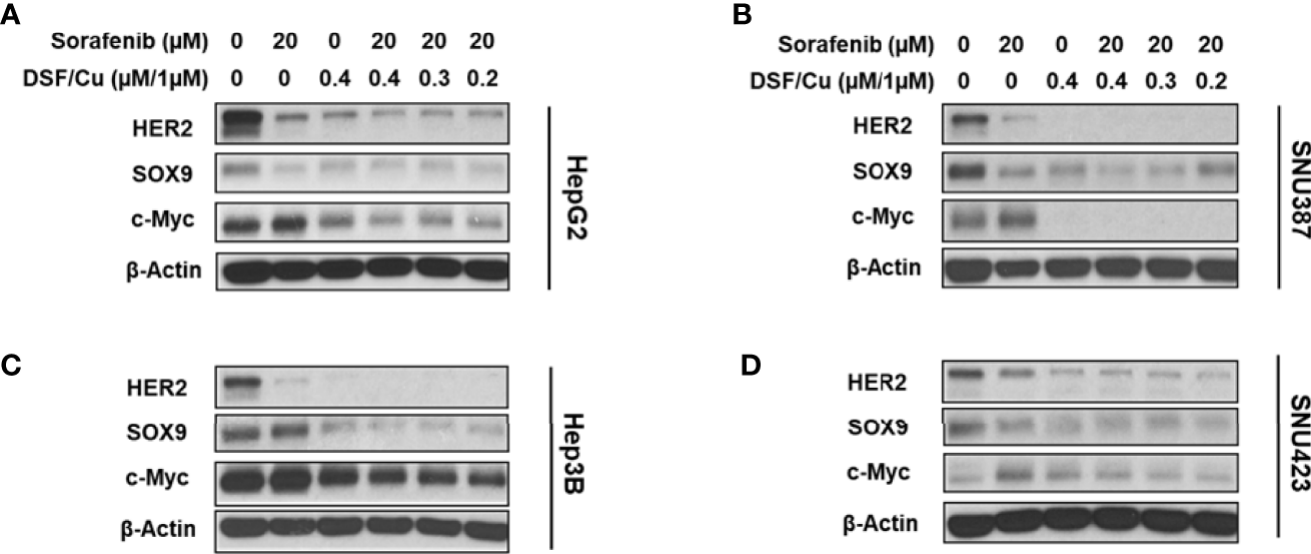
DSF/Cu and sorafenib decrease stemness gene expression in HCC cells. The HCC cell lines HepG2 **(A)**, SNU387 **(B)**, Hep3B **(C)**, and SNU423 **(D)** were treated as indicated for 24 h. Treated cells were lysed to detect stemness gene encoded protein expression by western blot analysis.

To examine the effect of DSF/Cu and sorafenib on stemness gene expression in HCC cells, the four HCC cell lines were treated with different doses and combinations of drugs and monitored for their expression of stemness genes at the protein level by western blot analysis. Sorafenib decreased HER2 and SOX9 expression but had little impact on c-Myc expression in all cell lines (**Figures 3A–D**). However, DSF/Cu alone or in combination with sorafenib decreased the expression of all three stemness genes to low or non-detectable levels (**Figures 3A–D**). These results indicate that DSF/Cu + sorafenib could effectively decrease the stemness of HCC cells *in vitro*, consistent with our previous studies in breast and pancreatic cancer cells (18, 27, 28).

### DSF/Cu and Sorafenib Induce Autophagy and Apoptosis of HCC Cells

**Figure 4:**
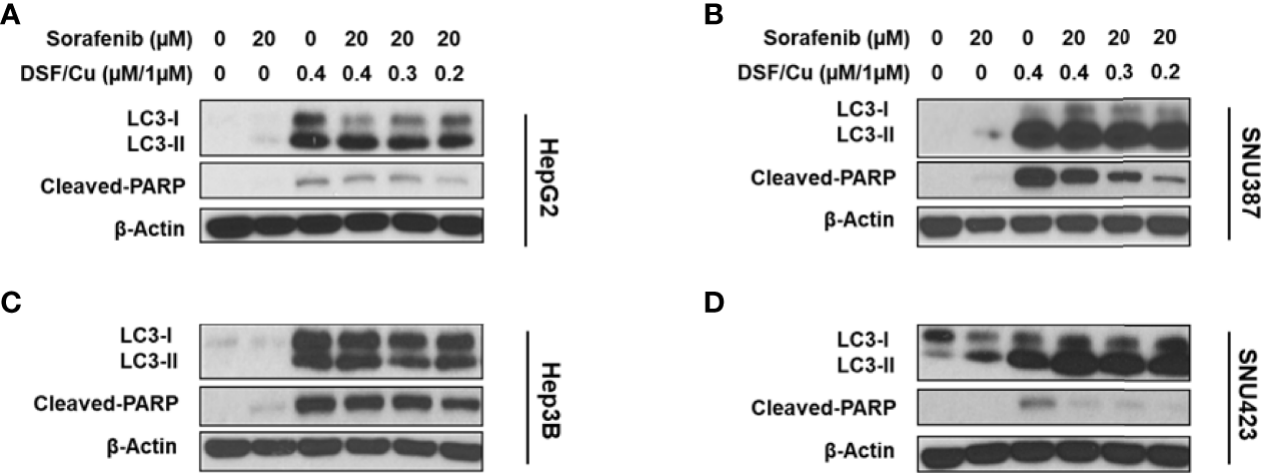
DSF/Cu and sorafenib induce autophagy and apoptosis of HCC cells. The HCC cell lines HepG2 **(A)**, SNU387 **(B)**, Hep3B **(C)**, and SNU423 **(D)** were treated as indicated for 24 h. Treated cells were lysed to detect levels of LC3-II/LC3-I and cleaved PARP by western blot analysis.

Previously, we demonstrated that DSF/Cu induces autophagic apoptosis in breast cancer and pancreatic ductal adenocarcinoma cell lines (25). The conversion of the cytosolic form of the microtubule-associated protein 1A/1B light chain 3B (LC3-I) to lipid-bound LC3-II is a marker of autophagy, whereas cleavage of poly (ADP-ribose) polymerase (PARP) by caspase-3 is a commonly used marker of apoptosis. To determine whether DSF/Cu + sorafenib could induce autophagy and apoptosis, cell lines were cultured in the presence or absence of DSF/Cu or sorafenib for 12 h followed by western blot analysis to determine LC3-II/LC3-I ratios, which reflect the amount of autophagy, and cleaved PARP levels. Whereas DSF/Cu-induced autophagy and apoptosis in all four cell lines, lower to non-detectable levels of LC3-II/LC3-I conversion and cleaved PARP were detected following treatment with sorafenib alone (**Figures 4A–D**). As expected, DSF/Cu + sorafenib enhanced the induction of autophagy and apoptosis in all four HCC cell lines in a dose-dependent manner as indicated by LC3-II/LC3-I ratios and cleaved PARP levels (**Figures 4A–D**). However, the results of autophagy and apoptosis must be interpreted with caution, as an in-depth study on cell death induced by DSF/Cu + sorafenib should be carried out with additional markers and methods (29).

**Figure 5:**
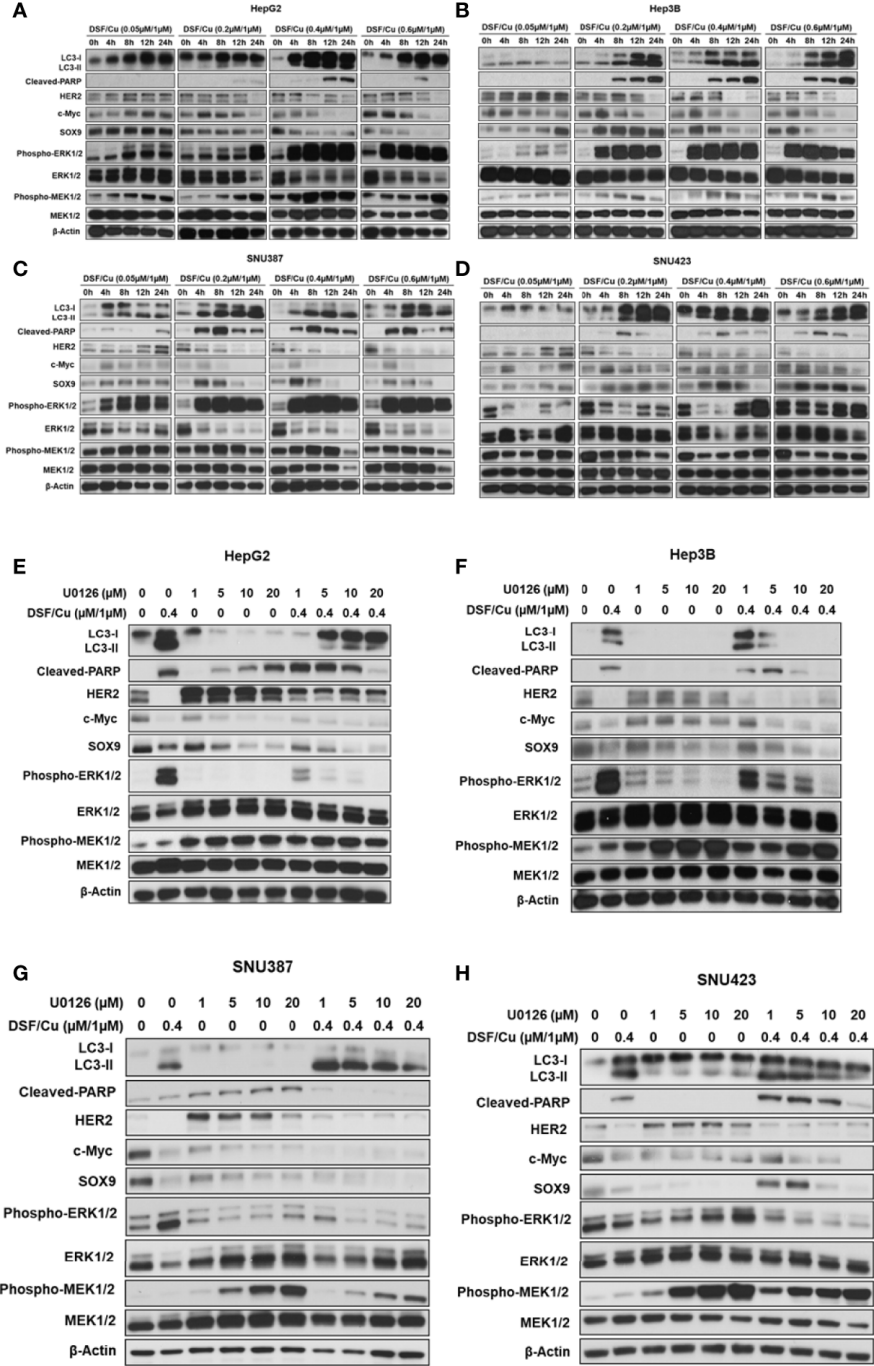
DSF/Cu induces autophagic cell death *via* sustained activation of the ERK signalling pathway *in vitro*. The HCC cell lines HepG2 **(A, E)**, Hep3B **(B, F)**, SNU387 **(C, G)**, and SNU423 **(D, H)** were treated with different doses of DSF/Cu in the presence or absence of U0126. Treated cells were lysed at different time points and analysed by western blot for the expression of stemness genes encoded proteins, autophagy, apoptosis markers, and MEK/ERK pathway activation-related genes.

The efficacy of sorafenib against advanced HCC is attributed to its direct inhibitory effects on the growth of HCC cells *via* the Raf/MEK/ERK signaling pathway and its indirect suppressive effects on HCC angiogenesis *via* inhibition of receptor tyrosine kinases, including VEGFR and PDGFR (5, 23). Given that DSF/Cu + sorafenib had a more potent anti-tumor effect than sorafenib alone, one hypothesis is that DSF/Cu functions synergistically with sorafenib by also inhibiting the MEK/ERK signaling pathway. To investigate the underlying mechanism involved in this process, the expression of MER/ERK pathway-related proteins in DSF/Cu-treated HCC cell lines was analyzed by western blot. Surprisingly, increased protein levels of phosphorylated ERK (p-ERK) and phosphorylated MEK (p-MEK) were detected in all four HCC cell lines in a dose- and time-dependent manner following DSF/Cu treatment (**Figures 5A–D**). However, a high dose (0.6 μM/1 μM) or a long period (24 h) of DSF/Cu treatment decreased expression of p-ERK and p-MEK in Hep3B (**Figure 5B**) and SNU387 (**Figure 5C**) cell lines. Moreover, high expression of MEK/ERK pathway-associated genes was generally associated with low protein expression of stemness, increased autophagy and apoptosis (**Figures 5A–D**) (**Supplementary Figures 1A–G**). These findings indicate that the autophagy, apoptosis and stemness of HCC cells were determined by sustained MEK/ERK activation induced by DSF/Cu.

To further confirm this mechanism, HCC cell lines were treated with U0126, a highly selective inhibitor of MEK1/2 and ERK activation, which decreased p-ERK levels (**Figures 5E–H**). However, U0126 increased p-MEK levels, possibly due to a negative feedback mechanism (30). In addition, U0126 induced apoptosis in HepG2 and SNU387 cell lines and autophagy in SNU387 and SNU423 cell lines (**Figures 5E–H**). Considering the expression of stemness genes, U0126 inhibition of ERK activation resulted in mixed responses in HCC cell lines, with increased HER2 expression and decreased SOX9 and c-Myc expression (**Figures 5E–H**). To investigate the function of DSF/Cu in the MEK/ERK signaling pathway in HCC cells, cells were exposed to DSF/Cu (0.4 μM/1 μM) and different doses of U0126 (1, 5, 10, or 20 μM). The inhibitor partially blocked DSF/Cu-induced conversion of LC3-I to LC3-II and PARP cleavage all four HCC cell lines. However, the effect of U0126 on DSF/Cu-induced downregulation of stemness gene encoded protein expression was not clearly noted (**Figures 5E–H**).

### The DSF With Endogenous Cu^2+^ and Sorafenib Is Significantly More Effective Than Sorafenib Alone in Inhibiting the Growth of Orthotopic HCC Xenografts in Mice

**Figure 6:**
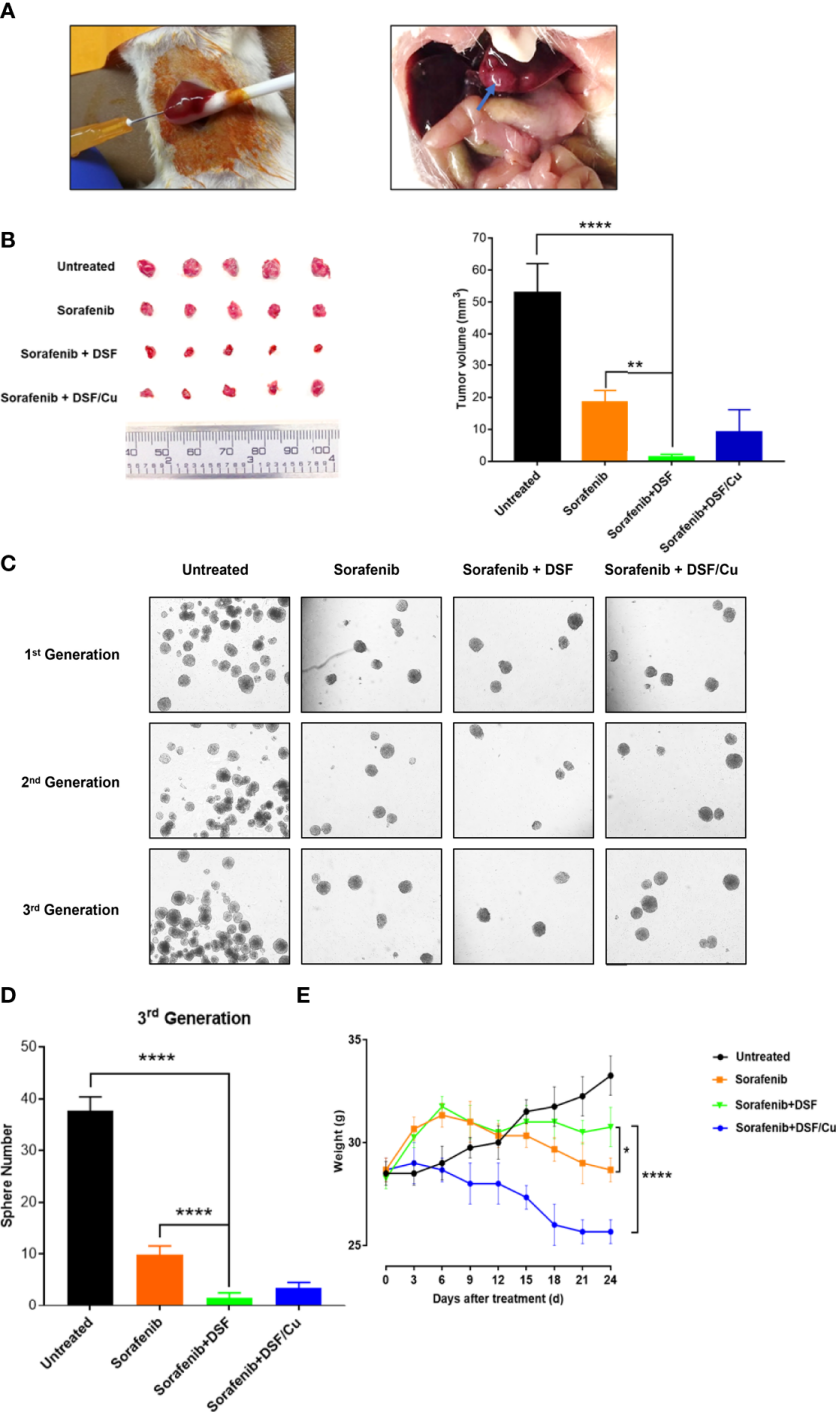
DSF with endogenous Cu^2+^ and sorafenib inhibits the growth of orthotopic HCC xenografts in mice more effectively than sorafenib alone. Human HepG2 cells were injected directly into the liver of NSG mice (left) and the resulting orthotopic liver tumor formation (right). **(A)**. Mice were grouped and all the treatments were initiated (on day 4). At the time of sacrifice (on day 24), tumors were resected and measured **(B)**. The sphere formation of cells isolated from the cells collected from xenograft tumors of each mouse group was assessed. Spheres were quantified by counting sphere numbers/well on day 14 **(C, D)**. To monitor toxicity, mouse body weight was measured every 3 days **(E)**. Data are shown as mean ± SD. *p<0.05, **p<0.01, ****p<0.0001.

To assess whether these *in vitro* findings could be extended to a preclinical animal tumor model, HepG2 cells were directly injected into the liver of immunodeficient NSG mice to establish orthotopic HCC xenografts (**Figure 6A**).

Mice treated with sorafenib + DSF had smaller tumors at day 24 than untreated (1.4 ± 0.78 mm vs. 52.9 ± 9.09 mm, p<0.0001) or sorafenib-treated (1.4 ± 0.78 mm vs. 18.55 ± 3.58 mm, p=0.0017) mice. Tumor size was similar between sorafenib + DSF and sorafenib + DSF/Cu groups, but there was a tendency toward reduced tumor volume in the sorafenib + DSF group compared with the sorafenib + DSF/Cu group (1.4 ± 0.78 mm vs. 9.56 ± 6.84 mm) (**Figure 6B**). Exogenous Cu^2+^ is not necessary for the efficacy of DSF in *in vivo* experiments due to the existence of endogenous Cu^2+^ in HCC (31) and other types of tumors (27). Sorafenib + DSF exerted a larger anti-tumor effect on third-generation exerted a larger anti-tumor effect on the third generationHCSCs, as evidenced by sphere formation compared with no treatment, sorafenib alone, or sorafenib + DSF/Cu (1.50 ± 1.05 vs. 36.67 ± 2.73, 9.83 ± 1.72, and 3.50 ± 1.05; p<0.0001, p<0.0001, and p=0.24, respectively) (**Figures 6C, D**). This finding suggests that sorafenib monotherapy only partially eliminates HCSCs, whereas sorafenib + DSF eliminates nearly all HCSCs. The interaction effect between DSF and sorafenib indicated a significant difference in terms of tumor volume and 3^rd^ generation of sphere numbers (p<0.0001).

As a preliminary gauge of the potential drug-induced toxicity of combined treatment, mouse body weight was measured every 3 days. The two-way ANOVA followed by the Tukey’s multiplicity comparison were used to determine the interaction between time and treatments (DSF), the results showed significant difference (p<0.0001). Notably, sorafenib alone and sorafenib + DSF/Cu groups showed greater loss of body weight than the sorafenib + DSF group (6.76% and 16.52% loss, p=0.034 and 0.0057, respectively) (**Figure 6E**). No DSF-associated drug toxicity was observed in in this mouse tumor model. On the contrary, DSF showed evidence of protection against sorafenib treatment-induced weight loss.

## Discussion

Considerable evidence indicates that HCSCs sustain tumor growth, produce differentiated progeny, and eventually result in tumor metastasis (32, 33). In addition to scientific consensus on their role in cancer is the finding that the presence of HCSCs is associated with poor prognosis and is predictive of clinical outcomes (34). Therefore, in this study, we aimed to develop an effective approach to targeting HCSCs to improve the efficacy of current chemotherapy for patients with advanced HCC.

The chemoresistance of HCSCs also extends to sorafenib (16). We found that sorafenib had a limited effect on HCSCs identified as ALDH^+^ HCC cells by flow cytometry or through their sphere formation ability. This could explain why many patients with advanced HCC are not sensitive to sorafenib. Increasing evidence from preclinical studies indicates that combining sorafenib with other drugs can enhance its anti-tumor efficacy (35, 36). However, poor clinical efficacy and undesirable side effects limit the clinical application of combined drug therapy in patients with advanced HCC (37, 38). Therefore, any successful method for improving cell sensitivity and reversing resistance to sorafenib has clinical potential.

DSF is an ALDH inhibitor that is shown to exert anti-tumor activity in multiple mouse syngeneic and xenograft tumor models and cancer patients (18, 27, 39–41). We previously demonstrated that DSF/Cu targets CSCs and thereby improves the efficacy of standard chemotherapy or radiotherapy in pancreatic ductal adenocarcinoma, breast cancer and chondrosarcoma (18, 27, 28, 41). The same conclusion was obtained in the present study using HCC cell lines by detecting the proportion of ALDH^+^ cells to identify HCSCs and assessing their sphere formation ability and expression of stemness genes HER2, SOX9, and c-Myc. Whereas exogenously supplied Cu^2+^ is needed for DSF to target CSCs and induce cancer cell apoptosis *in vitro* (18), exogenously supplied Cu^2+^ is not required *in vivo*. The liver plays an important role in the supply, storage, and secretion of Cu^2+^ (42). Compared with patients with benign liver diseases, HCC patients show much higher levels of serum Cu^2+^ (43), which are strongly associated with poor HCC-specific survival (44). In addition, compared with normal liver tissues or a primary hepatocyte line, HCC tissue or HCC tissues or HCC cell lines exhibit excessive accumulation of Cu^2+^ (45, 46). These facts may explain why the supplied exogenous Cu^2+^ reduced the anti-tumor effect of DSF accompanied by weight loss in the HCC mouse model used in this study, as Cu^2+^ at a higher dose, e.g., 5 µM is tumor-promoting (47) and Cu^2+^ is necessary for toxicity of DSF at concentrations < 4 μM (48). Relevantly, the high level of Cu^2+^ in liver may explain the failure of DSF and exogenous Cu^2+^ in a previous clinical trial for treatment of refractory solid tumors metastasized to liver (NCT00742911), suggesting that Cu^2+^ levels in tumor tissues and/or serum should be taken into consideration when conducting future clinical trials involving DSF.

The present study aimed to improve the efficacy of sorafenib for treating advanced HCC by repurposing DSF targeting HCSCs. As expected, DSF/Cu and sorafenib synergistically inhibited the growth of HCC cells *in vitro*. Compared with either drug used as monotherapy, the combination of DSF/Cu and sorafenib was more effective in eliminating HCSCs and induced more potent autophagy and apoptosis. The anti-tumor activity of sorafenib was attributed to its inhibition of angiogenesis (VEGFR and PDGFR) and direct effect on tumor cell proliferation and survival (RAF/MEK/ERK pathway) (23). Previous studies demonstrate that an MEK inhibitor combined with sorafenib synergistically exhibits anti-tumor activity (49, 50). In addition, some studies show that MEK inhibition can reduce the proliferation and self-renewal of CSCs in many tumor types, including HCC (51–53). Therefore, we suspected that DSF/Cu could serve as a MEK inhibitor to improve the anti-tumor effect of sorafenib. Surprisingly, however, DSF/Cu was found to activate the ERK/MEK signaling pathway and sustain high expression of p-ERK and p-MEK, which was associated with the loss of stemness gene expression. This finding is contrary to those of previous studies, which suggest that MEK inhibition contributes to the apoptosis of HCC cells and HCSCs (23, 51). Furthermore, substantial evidence indicates that high expression of p-ERK may serve as a good prognostic biomarker for sorafenib response in HCC (54, 55). The synergistic effect of DSF/Cu + sorafenib on HCC can be interpreted by the fact that DSF/Cu maintains activation of ERK/MER pathway, which is harmful to HCC cells and HCSCs, and elevates p-ERK, thereby improving the sensitivity of HCC to sorafenib. It is important to further study the mechanisms of DSF/Cu + sorafenib on bulk HCC cells and sorted HCSCs. Also, we plan to establish sorafenib-resistant HCC cell lines to further reveal the mechanisms involved.

To generate more clinically relevant and translational data on DSF/Cu + sorafenib therapy, we established an orthotopic HCC xenograft model in mice and found that combination therapy was significantly more effective in inhibiting xenografts than monotherapy. Of note, DSF did not cause additional toxicity but appeared to have a protective role, consistent with our previous results in pancreatic ductal adenocarcinoma and breast cancer mouse models (18, 27). Together, these *in vitro* and *in vivo* findings provide a foundation for further developing an effective clinical strategy involving the repurposing of DSF to enhance the therapeutic efficacy of sorafenib against HCC.

## Data Availability Statement

The original contributions presented in the study are included in the article/**Supplementary Material**. Further inquiries can be directed to the corresponding author.

## Ethics Statement

The animal study was reviewed and approved by The Institutional Animal Care and Use at the Massachusetts General Hospital.

## Author Contributions

XW conceived and designed this study. GZ, YW, WG, LC, DE, and LZ collected the data. GZ, YW, XW, BF, DE, HZ, and KT analysed and interpreted the data. YW, XW, AD and DD, were involved in writing, revision of the manuscript. All authors contributed to the article and approved the submitted version.

## Funding

This work was supported by grants R01CA226981(XW) and W81XWH-20-PCRP-IDA (W81XWH2110433, XW).

## Conflict of Interest

The authors declare that the research was conducted in the absence of any commercial or financial relationships that could be construed as a potential conflict of interest.

## Publisher’s Note

All claims expressed in this article are solely those of the authors and do not necessarily represent those of their affiliated organizations, or those of the publisher, the editors and the reviewers. Any product that may be evaluated in this article, or claim that may be made by its manufacturer, is not guaranteed or endorsed by the publisher.

## Supplementary Material

Supplementary Figure 1 DSF/Cu downregulates the expression of stemness gene encode proteins and induces autophagic cell death *via* sustained activation of the ERK signaling pathway. The HCC cell lines HepG2, Hep3B, SNU387, and SNU423 were treated with DSF/Cu (0.4µM/1µM). Treated cells were lysed at different time points and analyzed by western blot for the expression of stemness genes encoded proteins, autophagy, apoptosis markers, and MEK/ERK pathway activation-related genes. The band density of each protein was quantified with ImageJ. The mean ± SD of each protein from 2 independent blots (while the protein expression level was relative to 0 h time point) are shown. * Indicates p<0.05, ** indicates p<0.01, *** indicates p<0.001, and **** indicates p<0.0001.
