# Correction: Improving the therapeutic efficacy of sorafenib for hepatocellular carcinoma by repurposing disulfiram

**DOI:** 10.3389/fonc.2026.1772315

**Published:** 2026-02-18

**Authors:** Gong Zhang, Yufeng Wang, Bryan C. Fuchs, Wei Guo, David L. Drum, Derek J. Erstad, Baomin Shi, Albert B. DeLeo, Hui Zheng, Lei Cai, Liyuan Zhang, Kenneth K. Tanabe, Xinhui Wang

**Affiliations:** 1 Division of Gastrointestinal and Oncologic Surgery, Department of Surgery, Massachusetts General Hospital, Harvard Medical School, Boston, MA, United States; 2 Department of Hepatobiliary and Pancreatic Surgery, The First Affiliated Hospital of Zhengzhou University, Zhengzhou, Henan, China; 3 Department of General Surgery, Tongji Hospital, School of Medicine, Tongji University, Shanghai, China; 4 Biostatistics Center, Massachusetts General Hospital, Harvard Medical School, Boston, MA, United States

**Keywords:** sorafenib, disulfiram, copper, hepatic cancer stem cells, ERK pathway

There was a mistake in **Figure 2E** as published. Some of the sphere images were unintentionally duplicated during the preparation process. The corrected **Figure 2** and its caption appear below.

**Figure 2:**
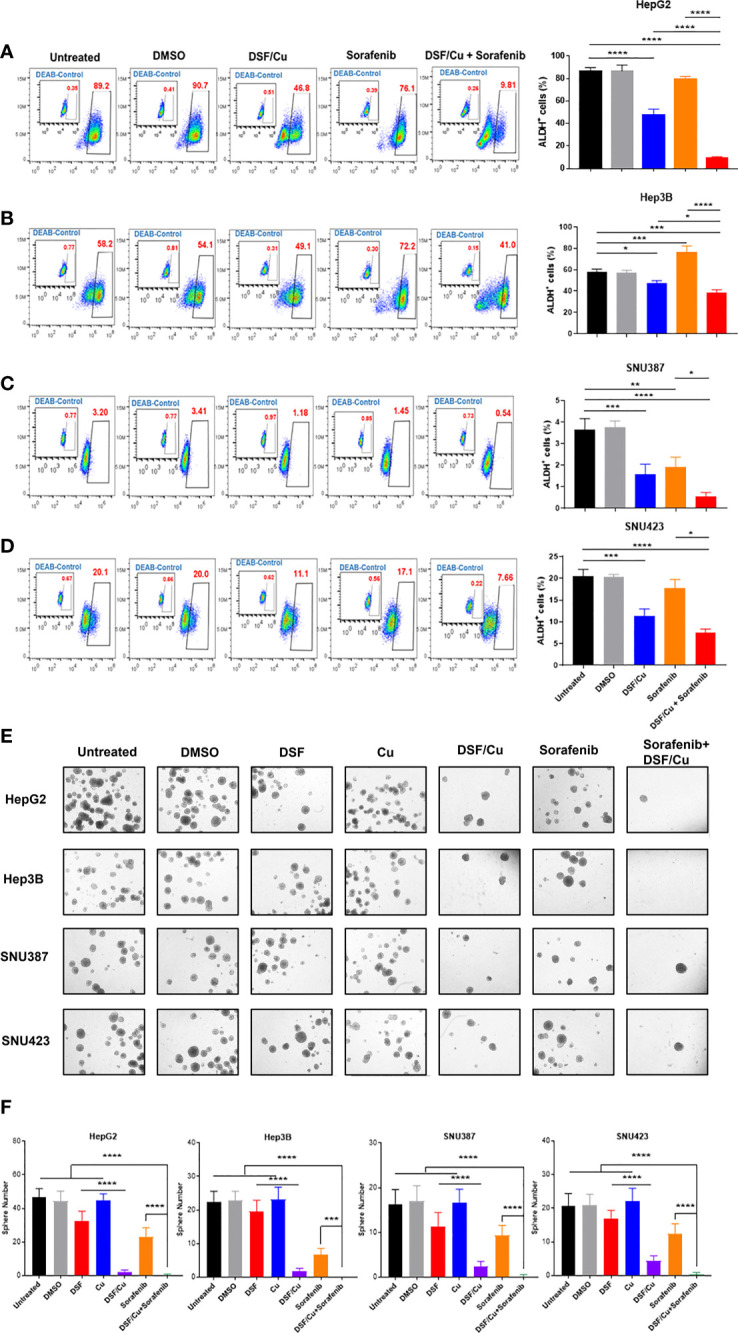
HCSCs are sensitive to DSF/Cu and sorafenib. The HCC cell lines HepG2 **(A)**, Hep3B **(B)**, SNU387 **(C)**, and SNU423 **(D)** were treated as indicated, and ALDH+ cells was measured using flow cytometry. Sphere formation assays were performed in six-well plates by seeding treated cells and culturing for 14 days. Spheres were quantified by counting sphere numbers per well on day 14 **(E, F)**. All experiments were performed in triplicate for each cell line, and data are shown as mean ± SD. *p<0.05, **p<0.01, ***p<0.001, ****p<0.0001.

There was a mistake in **Figure 6C** as published. Some of the sphere images were unintentionally duplicated during the preparation process. The corrected **Figure 6** and its caption appear below.

**Figure 6:**
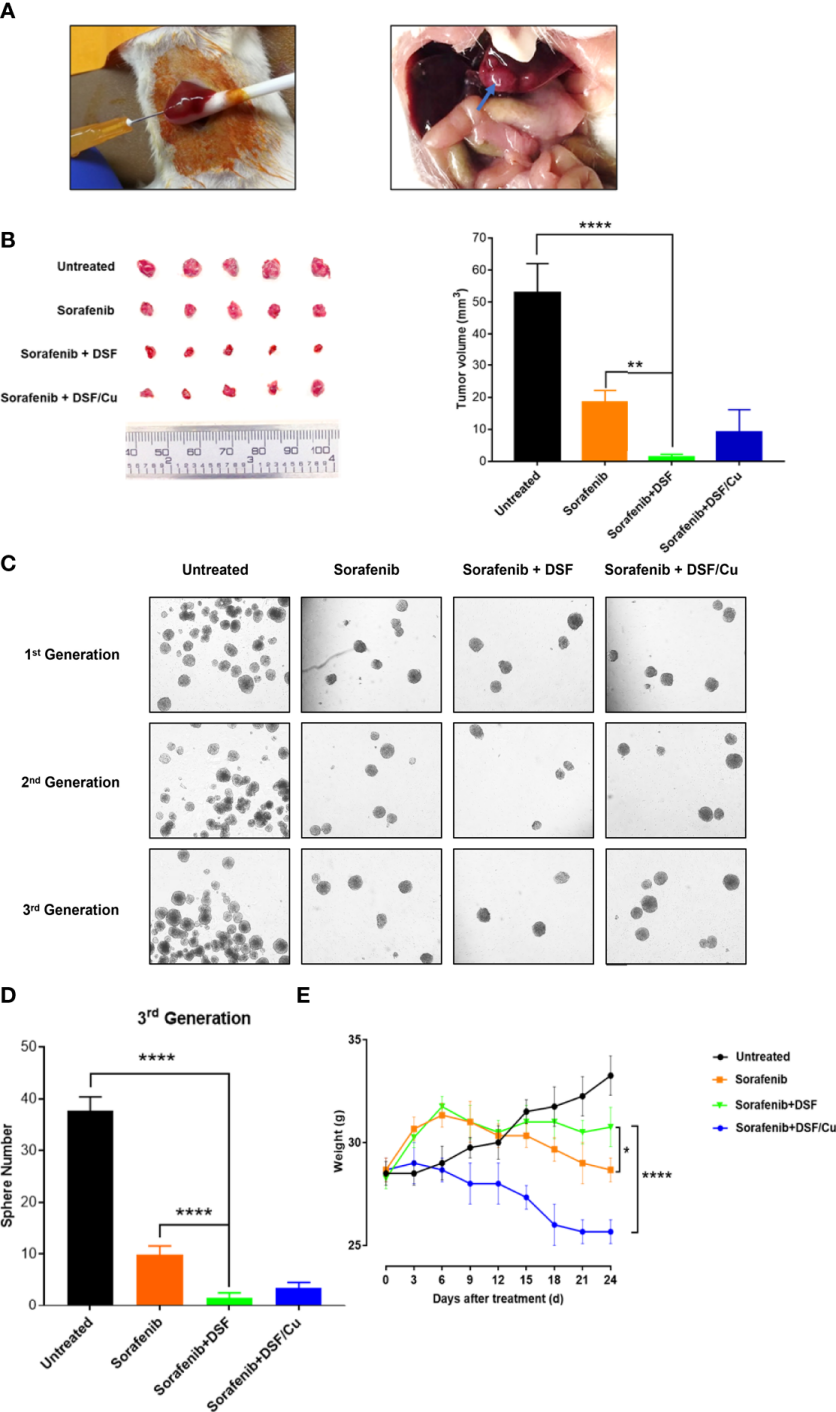
The sphere formation of cells isolated from the cells collected from xenograft tumors of each mouse group was assessed. Spheres were quantified by counting sphere numbers/well on day 14 **(C, D)**.

The original version of this article has been updated.

