# Supplementary figures and images for: Improving the Therapeutic Efficacy of Sorafenib for Hepatocellular Carcinoma by Repurposing Disulfiram
Source: Front Oncol. 2022 Jul 14;12:913736. doi: 10.3389/fonc.2022.913736 (PMC9329590; doi:10.3389/fonc.2022.913736)

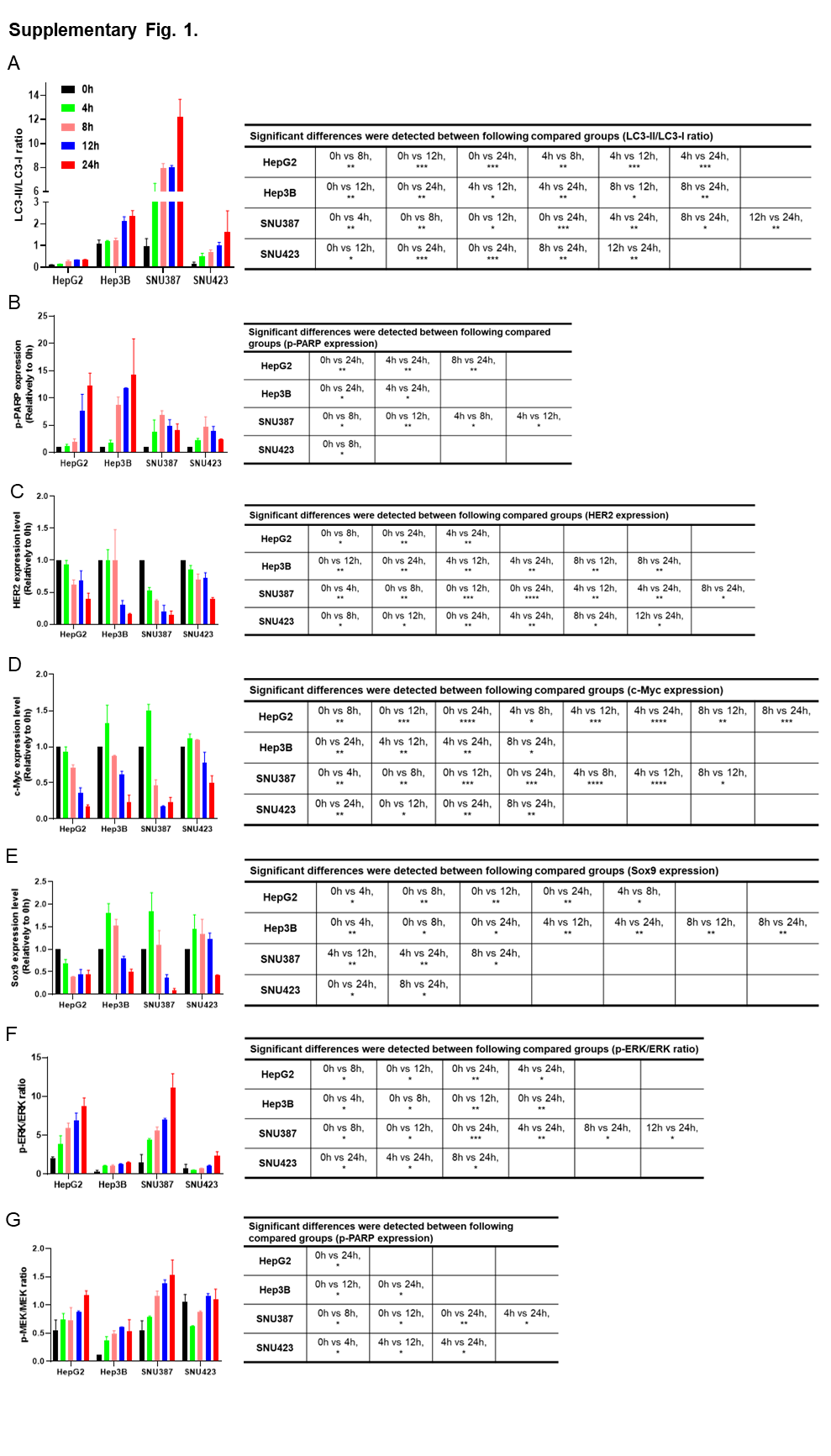

Supplement: Supplementary Figure 1 — DSF/Cu downregulates the expression of stemness gene encode proteins and induces autophagic cell death via sustained activation of the ERK signaling pathway. The HCC cell lines HepG2, Hep3B, SNU387, and SNU423 were treated with DSF/Cu (0.4µM/1µM). Treated cells were lysed at different time points and analyzed by western blot for the expression of stemness genes encoded proteins, autophagy, apoptosis markers, and MEK/ERK pathway activation-related genes. The band density of each protein was quantified with ImageJ. The mean ± SD of each protein from 2 independent blots (while the protein expression level was relative to 0 h time point) are shown. * Indicates p<0.05, ** indicates p<0.01, *** indicates p<0.001, and **** indicates p<0.0001. [file Image1.tif]
